# Vegetation restoration of abandoned cropland improves soil ecosystem multifunctionality through alleviating nitrogen-limitation in the China Danxia

**DOI:** 10.3389/fpls.2023.1116179

**Published:** 2023-02-28

**Authors:** Chao Wang, Qiannan Yang, Chi Zhang, Xiaolong Zhang, Jing Chen, Kexue Liu

**Affiliations:** ^1^ School of Resources and Planning, Guangzhou Xinhua University, Guangzhou, China; ^2^ Ecological Restoration Research Center, China Institute of south China Urban-Rural Economic and Social Development, Guangzhou, China; ^3^ College of Natural Resources and Environment, South China Agricultural University, Guangzhou, China

**Keywords:** soil enzyme activity, enzyme stoichiometry, microbial nutrient limitation, soil ecosystem multifunctionality, vegetation restoration, China Danxia

## Abstract

The microbial requirement for nutrient resources can be estimated by soil extracellular enzyme stoichiometry (EES) and their stoichiometries. Implementing the Grain for Green Program has significantly impacted land use and soil nutrient management in the China Danxia. However, drivers of soil microbial nutrient limitation changes in abandoned cropland (AC) remained unclear after vegetation restoration. Here, according to vector analysis, we evaluated microbial nutrient limitation by studying soil EES across vegetation restoration types (naturally restored secondary forests (NF) and artificially planted forests (AF)) with AC as a control. Results showed both NF and AF soils averaged higher C- and P- acquiring enzyme, indicating rapid C and P turnover rates after vegetation restoration. However, vegetation restoration resulted in higher C requirement for microorganisms with higher enzyme C:N and vector length. In addition, microorganisms shifted from N- (< 45°) to P-limited (> 45°) conditions with enzyme N:P less than 1 after vegetation restoration, and NF exacerbated microbial P limitation compared to AF. Decreased N limitation following vegetation restoration could be contributed to improving soil ecosystem multifunctionality. The greater variation of EES was explained by the interaction of pH, soil nutrient, and microbial biomass than by any one of these factors alone, suggesting that both abiotic and biotic factors regulate microbial nutrient limitation and microbial process. Overall, our results revealed vegetation restoration could alleviate N limitation in the China Danxia, and thus enhance soil ecosystem by regulating lower microbial N limitation, which provide insight into nutrient management strategies under ecological restoration of degraded areas.

## 1 Introduction

Agricultural overexpansion has degraded ecosystem functions and destroyed land productivity in some forested areas with poor site conditions (Santos et al., 2021), resulting in large-scale farmland abandonment. From 1992–2015, 83 million hectares of cropland were abandoned globally, equivalent to 5 percent of today’s arable land (Naess et al., 2021). China implemented the Grain for Green Program in the 1990s, which has converted a large amount of sloping and abandoned cropland to grasslands and forests (Wang et al., 2021). The restoration process of abandoned cropland changes the original vegetation type and pattern (Gu et al., 2021) while improving the soil microbial community structure (Zhong et al., 2020). Additionally, this process effectively improves the regional site environment and influences the nutrient balance of terrestrial ecosystems (Knelman et al., 2015). However, as both above- and below-ground biotic communities growing, uneven nutrient availability and plant-microbe competition for nutrients lead to soil nutrient limitation, which negatively impacts soil quality maintenance and ecosystem stability (Bruggen et al., 2019; Deng et al., 2019). Therefore, insight into the status and influencing factors of soil nutrient limitation in abandoned cropland is vital for assessing soil nutrient availability and guiding soil nutrient management during vegetation restoration in ecologically degraded areas.

Soil extracellular enzyme activities (EEA) are direct mediators of organic matter decomposition, controlling soil material cycling and energy flow (Blonska et al., 2021). EEA are quick to react to changes in environmental factor, especially in soil nutrients (Cui et al., 2021). Insufficient soil nutrient availability often imposes energy and nutrient constraints on microbial growth and metabolism, which in turn affects the microbial expression of EEA (Chen et al., 2019). The optimal allocation model in ecological economics states that microorganisms can commit more resources to acquire limiting nutrients (Treseder and Vitousek, 2001; Sinsabaugh et al., 2008). Extracellular enzyme stoichiometry (EES) is a measure of equilibrium of availability and microbial requirements for nutrients (Sinsabaugh et al., 2008; Sinsabaugh et al., 2009). Globally, the enzymatic C:N:P is highly constrained, converging on nearly 1:1:1 after logarithmic transformation (Sinsabaugh et al., 2008). However, as soil microorganisms secrete EEA in order to obtain limiting nutrients for growth and metabolism, EES will be altered inevitably (Sinsabaugh et al., 2009; Cui et al., 2021). Yang et al. (2020) reported that on the different stages of the restoration of loess plateau grasslands, the regional EES was 1:1.08:1.28 and N and P co-limited microorganisms. Guan et al. (2022) noted that while vegetation restoration exacerbated N shortage in abandoned cropland, it boosted activities of enzyme related C and N uptake and decreased enzymatic C:N in karst environments. As shown above, both EEA and EES are sensitive indicators of the extent to which microorganisms are energy- and nutrient-limited and may provide a new perspective for evaluating soil nutrient limitation in abandoned cropland.

The China Danxia refers to a red terrestrial landscape of sedimentary origin formed by endogenous (crustal uplift) and exogenous (weathering and water erosion) forces, covering an area of about 822 km^2^ (UNESCO, 2010). The China Danxia is a particularly ecologically fragile region in South China because of its prolonged exposure to exogenous forces, which resulted in its soils losing ions and nutrient elements and becoming acidic to strongly acidic, with a loose physical structure, thin soil layers, and exposed parent material (Peng et al., 2011; Yan and Kasanin-Grubin, 2019). Consequently, the Grain for Green Program has been implemented widely in South China due to land degradation in the China Danxia (Yan and Kasanin-Grubin, 2019). Vegetation restoration has been shown to be an effective practice in reverse the declining trend of land productivity by improving a variety of ecosystem services and functions (Yang et al., 2020; Resch et al., 2021). However, most previous studies had focused only on soil nutrients, soil enzyme activities, or soil microbial composition, with little information on microbial metabolic limitations and comprehensive soil ecosystem.

In this study, a comparison of microbial metabolism characteristics and soil multifunctionality under different vegetation restoration types were explored using enzyme stoichiometry for naturally restored secondary forests (NF) and artificially planted forests (AF) in the soil degradation area of China Danxia. Abandoned cropland (AC) without vegetation restoration was used as a control. Specifically, our study aimed to: (1) compare NF and AF in soil EEA and soil ecosystem multifunctional (EMF) to AC; (2) distinguish abandoned cropland from vegetation restoration in terms of microbial nutrient limitation; (3) analyse whether microbial nutrient limitation affect soil EMF; (4) reveal how changes in environmental factors affect microbial resource limitation. We hypothesized that soil microbial resources limitation and soil multifunctionality may be significantly influenced following vegetation restoration. Nitrogen as early limiting element in soils and may be a significant factor in limiting increased soil multifunctionality. We also hypothesized that the limitation of soil microbial resources may be controlled by a combination of biotic and abiotic factors and mainly influenced by soil nutrients and soil microbial biomass.

## 2 Materials and methods

### 2.1 Study area

The study region lies within the Danxia Mountain World Geopark in Renhua County, Shaoguan City, Guangdong Province (24°51′48″–25°04′12″N, 113°36′25″–113°47′53″E), which is in the transition area of the mid-subtropical zone to the southern subtropical zone with an average altitude of 220 m, a mean annual temperature of 19.9 °C and a precipitation of 1,512.3 mm. Its geology is characterised by the China Danxia composed of red glutenite layers, with Alfisols (FAO-UNESCO, 1974) as the main soil type. The extensive reclamation and farming of forest land in the 1980s caused a sharp decrease in forests and the degradation of ecosystems with widespread soil erosion. The Grain for Green Program’s implementation and the World Geopark led to the research area eventually becoming a site for ecological restoration. *Capsicum annuum* L. and *Zea mays* L. were the primary crops planted on the cropland prior to abandonment, and the abandonment was believed to have lasted for 2–3 years, according to local government documents (Renhua County local Chronicles compilation Committee, 2014). Two types of restored vegetation were investigated in this study, namely the NF vegetation (> 30 years of age) dominated by *Firmiana danxiaensis* H. H. Hsue & H. S. Kiu and *Cinnamomum burmannii* (Nees et T.Nees) Blume, and the AF vegetation (> 30 years of age) dominated by *Cunninghamia lanceolata* (Lamb.) Hook and *Bombax ceiba* L., in comparison with AC. Areas with similar stand conditions, land use history, and soil types were selected to comprise the study region to reduce the influence of external conditions on experimental results.

### 2.2 Soil sample collection

In 2021, three 10 m × 10 m plots were randomly selected in a representative sampling field of each of the three ecosystems. Five sampling sites were set up along “X” shaped area in each plot, and soil samples were collected from both 0–10 and 10–20 cm soil layers using a soil auger. After removing plant residues and large gravels, the soil samples collected in the same depth layer from the five sampling sites of the same plot were mixed well. Fresh soil samples were passed through 2-mm sieve to determine microbial biomass and extracellular enzyme activities. The part of soil samples was naturally air-dried and then passed through 2- and 0.15- mm sieves for subsequent determination of soil pH and nutrient contents.

### 2.3 Sample analysis and measurement

Soil pH was determined using a pH potentiometer in deionized water (soil–water ratio 1:2.5, w/v) (Lu, 2000); soil total carbon (SOC), nitrogen (STN) and phosphorus (STP) were measured *via* potassium dichromate oxidation–reduction method, the Kjeldahl method, and perchloric acid–hydrofluoric acid digestion procedure method, respectively (Lu, 2000); microbial biomass carbon (MBC) and nitrogen (MBN) contents were detected using the chloroform fumigation-potassium sulfate leaching method with a leaching factor of 0.45 and 0.54, respectively (Lu, 2000); and microbial biomass phosphorus (MBP) content was determined using the chloroform fumigation-sodium bicarbonate leaching method with a leaching factor of 0.40 (Gai et al., 2021).

The activity of six extracellular enzymes β-1,4-glucosidase (BG), cellobiohydrolase (CBH), β-1,4-xylosidase (BX), β-1,4-acetylaminoglucosidase (NAG), leucine aminopeptidase (LAP), and alkaline phosphatase (AP) was determined by a 96-well fluorometric plate reader (Spectra Max M5, Molecular Devices Co., LTD, Shanghai) (Sinsabaugh et al., 2008; Yang et al., 2020). The measured EEA were calculated as the number of moles of enzyme per gram of soil sample per hour, and their corresponding substrates were found in **Table S1**.

### 2.4 Statistical analysis

EEA vector analysis was used to quantify soil microbial nutrient limitation using the following formulas (Moorhead et al., 2016):


X=(BG + CBH + BX)/[(BG + CBH + BX)+ AP]

Y=(BG + CBH + BX)/[(BG + CBH + BX)+(NAG + LAP)]

$$Vector\;length=SQRT(X^{2}+Y^{2})$$

Vector angle=DEGREES((ATAN2(X; Y)))

where vector length indicates the extent to which microorganisms are C-limitation, with a vector angle< 45° and > 45° indicating that soil microorganisms are N- and P-limitation, respectively.

The soil microbial stoichiometric homeostasis (*1/H*) is calculated using the following regression equation


Lny=c + 1/H Lnx

where *1/H* is the slope, *y is* the nutrient stoichiometry of soil microbial biomass, *x* is the nutrient stoichiometry of soils, and *c* is a constant. When *1/H is* 0, the regression is insignificant, and microorganisms are considered to have strong stoichiometric homeostasis. The regression is significant in the case of 0< *1/H<* 0.25, 0.25< *1/H<* 0.5, 0.5< *1/H<* 0.75, or *1/H* > 0.75, when the microorganisms are “homeostatic”, “weakly homeostatic”, “weakly plastic”, or “plastic”, respectively (Persson et al., 2010; Xiao et al., 2021).

For soil EMF, 12 soil function indicators (C: SOC, MBC, BG, CBH, BX, N: STN, MBN, NAG, LAP, P: STP, MBP, and AP) were selected and subjected to Z-standardization (Delgado-Baquerizo et al., 2016; Ma et al., 2022), with the mean value of the standardised indicators representing soil EMF (Wagg et al., 2014).

Statistical analysis was performed using SPSS 24.0 (SPSS Inc., Chicago, IL, USA). All variables were analysed using one-way and two-way ANOVA, and difference significance was tested using the LSD method (*p<* 0.05). Values are mean ± standard deviation (SD). The path whereby soil factors affect EMF was identified using partial least squares path modelling (PLS-PM) in the R 4.2.1 *plspm* package. The relationship between soil nutrients, microbial biomass, and EEA was evaluated using the Mantel test. The effects of soil nutrients and microbial biomass on soil EES were evaluated using variance partitioning analysis (VPA). Mantel test, and VPA were performed using the *Vegan* package of R 4.2.1. Plots were generated using Origin 2020b.

## 3 Results

### 3.1 Soil physicochemical properties

Vegetation restoration significantly changed soil physicochemical properties (**Table 1**). The pH, STN, STP, N:P, MBN, MBP, and N:P_mic_ of NF and AF soils were significantly higher than those of AC soils. In particular, NF soils exhibited significantly higher pH, SOC, STN, C:P, MBN, and MBP than the other two groups of soils. NF soils exhibited significantly higher C:N and MBC than AF soils but did not differ significantly from AC soils. Both C:N_mic_ and C:P_mic_ were highest in AC soils, with no significant differences between NF and AF soils.

**Table 1:** Soil physicochemical properties for each vegetation restoration type.

Indicator	Unit	AC	NF	AF	*F*
pH		5.23 ± 0.13c	6.21 ± 0.11a	5.96 ± 0.10b	142.79^***^
SOC	g kg^-1^	10.82 ± 2.65b	17.14 ± 3.17a	11.60 ± 3.17b	275.95^***^
STN	g kg^-1^	0.73 ± 0.10c	1.41 ± 0.42a	1.25 ± 0.18b	152.56^***^
STP	g kg^-1^	0.43 ± 0.10b	0.63 ± 0.16a	0.60 ± 0.10a	324.02^***^
C:N		14.62 ± 2.17a	12.60 ± 2.03a	9.16 ± 1.29b	34.95^***^
C:P		25.27 ± 1.05b	27.61 ± 2.12a	18.94 ± 2.25c	106.39^***^
N:P		1.76 ± 0.26b	2.21 ± 0.18a	2.07 ± 0.12a	14.28^***^
MBC	mg kg^-1^	119.77 ± 41.04a	120.04 ± 64.33a	97.11 ± 63.91b	15.42^***^
MBN	mg kg^-1^	9.07 ± 2.27c	28.22 ± 13.55a	18.29 ± 9.68b	108.95^***^
MBP	mg kg^-1^	16.44 ± 4.02c	28.75 ± 1.35a	19.58 ± 3.72b	161.48^***^
C:N_mic_		13.44± 3.87a	4.10 ± 0.49b	4.95 ± 1.01b	262.77^***^
C:P_mic_		7.36 ± 2.09a	4.13 ± 2.11b	4.75 ± 3.02b	81.97^***^
N:P_mic_		0.55 ± 0.03b	0.97 ± 0.43a	0.90 ± 0.45a	40.67^***^

Values are mean ± SD. AC, abandoned cropland; NF, naturally restored secondary forest; AF, artificial planted forest; SOC, soil organic carbon; STN, soil total nitrogen; STP, soil total phosphorus; C:N, ratio of SOC to STN; C:P, ratio of SOC to STP; N:P, ratio of STN to STP; MBC, microbial biomass carbon; MBN, microbial biomass nitrogen; MBP, microbial biomass phosphorus; C:N_mic_, ratio of MBC to MBN; C:P_mic_, ratio of MBC to MBP; N:P_mic_, ratio of MBN to MBP. Different lowercase letters indicated significant differences between vegetation restoration types at P< 0.05. supplement ***P < 0.001.

### 3.2 Changes in soil EEA and EES

**Figure 1:**
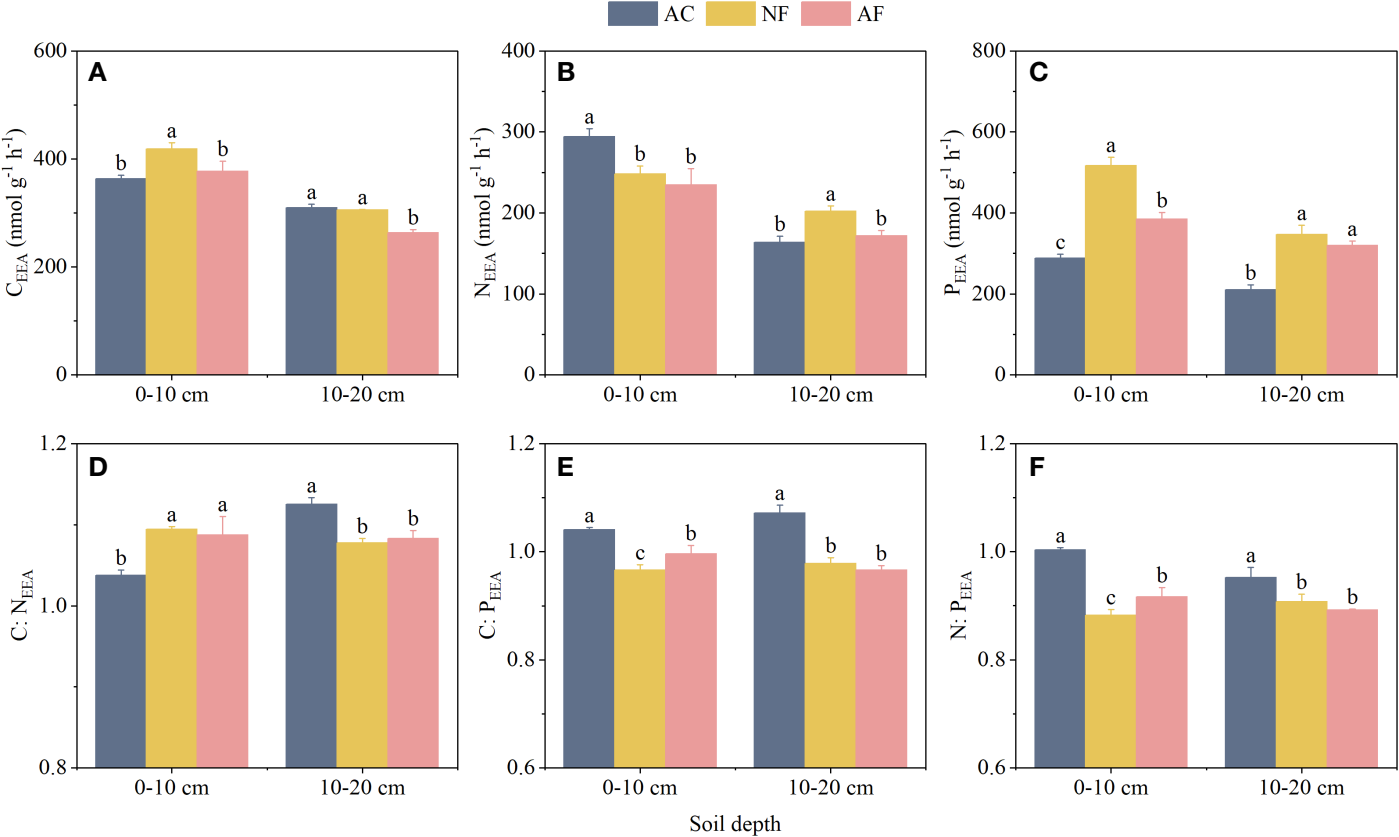
Effect of vegetation restoration on soil EEA and EES in abandoned cropland. **(A)** C_EEA_; **(B)** N_EEA_; **(C)** P_EEA_; **(D)** C_EEA_ : N_EEA_; **(E)** C_EEA_ : P_EEA_; **(F)** N_EEA_ : P_EEA_. AC, abandoned cropland; NF, naturally restored secondary forest; AF, artificial planted forest; CEEA, the sum of β-1,4-glucosidase, β-Dcellobiohydrolase and xylosidase; NEEA, the sum of β-N-acetylglucosaminidase and leucine aminopeptidase; PEEA, alkaline phosphatase. Different lowercase letters indicated significant differences between vegetation restoration types (LSD, *P***<** 0.05).

Soil C_EEA_, N_EEA_, and P_EEA_ were significantly affected by vegetation restoration (**Figures 1A–C**). At the 0–10 cm soil depth, C_EEA_ and P_EEA_ were significantly increased by 15.16% and 79.10%, respectively, in NF soils compared to AC soils, while N_EEA_ was significantly decreased in NF and AF soils. At the 10–20 cm soil depth, N_EEA_ and P_EEA_ were significantly increased by 23.71% and 64.48%, respectively, in NF soils, and P_EEA_ was significantly increased by 51.53% in AF soils compared to AC soils. In addition, soil depth also significantly affected soil C_EEA_, N_EEA_, and P_EEA_ (**Table 2**).

**Table 2:** ANOVA results for soil EEA and EES as affected by vegetation restoration (VR), soil depth (SD), and their interactions (VR × SD).

Indicator	Vegetation restoration (VR)		Soil depth (SD)		VR × SD	
	*F*	*P*	*F*	*P*	*F*	*P*
C_EEA_	27.259	< 0.001	403.602	< 0.001	18.416	< 0.001
N_EEA_	9.466	0.003	235.667	< 0.001	24.707	< 0.001
P_EEA_	196.427	< 0.001	191.616	< 0.001	18.915	< 0.001
C:N_EEA_	0.722	0.506	20.056	< 0.001	49.389	< 0.001
C:P_EEA_	103.409	< 0.001	1.136	0.307	12.864	< 0.001
N:P_EEA_	79.321	< 0.001	8.036	0.015	14.893	< 0.001
Vector length	34.634	< 0.001	14.779	0.002	46.084	< 0.001
Vector angle	97.668	< 0.001	1.320	0.273	13.494	< 0.001

C_EEA_, the sum of BG, CBH, and BX; N_EEA_, the sum of NAG and LAP; P_EEA_, AP activity; C:N_EEA_, Ln(BG + CBH + BX):Ln(NAG + LAP); C:P_EEA_, Ln(BG + CBH + BX):Ln(AP); N: P_EEA_, Ln(NAG + LAP):Ln(AP).

Compared to AC soils, vegetation restoration significantly increased C:N_EEA_ and significantly decreased C:P_EEA_ and N:P_EEA_ at the 0–10 cm soil depth of NF and AF soils (**Figures 1D–F**). In contrast, vegetation restoration significantly decreased C:N_EEA_, C:P_EEA_, and N:P_EEA_ at the 10–20 cm soil depth of NF and AF soils compared to AC soils. In addition, soil depth significantly affected C:N_EEA_, while it had no significant effect on C:P_EEA_ and N:P_EEA_ (**Table 2**).

### 3.3 Soil microbial metabolic limitation

**Figure 2:**
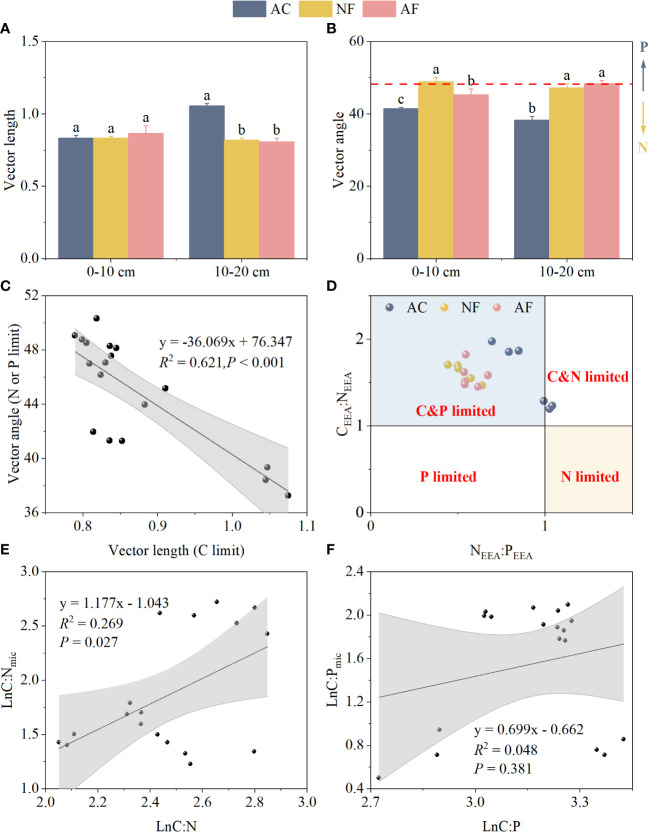
Effect of vegetation restoration on soil vector characteristics and microbial homeostasis in abandoned cropland. **(A)** vector length; **(B)** vector angle; **(C)** the relationship of vector length and angle; **(D)** microbial resource limitation; **(E)** microbial community homeostasis related to N; **(F)** microbial community homeostasis related to P. AC, abandoned cropland; NF, naturally restored secondary forest; AF, artificial planted forest; C_EEA_, the sum of BG, CBH, and BX; N_EEA_, the sum of NAG and LAP; P_EEA_, AP activity; C:N, ratio of SOC to STN; C:P, ratio of SOC to STP; C:N_mic_, ratio of MBC to MBN; C:P_mic_, ratio of MBC to MBP. Different lowercase letters indicated significant differences between vegetation restoration types (LSD, *P***<** 0.05).

Vector length at the 0–10 cm soil depth was not significantly affected by vegetation restoration. The vector length changed significantly from 1.06 to 0.81 at the 10–20 cm soil depth, and decreased after vegetation restoration, which indicated the microbial C-limitation weakened gradually (**Figure 2A**). Compared with AC, both NF and AF exhibited vector angles > 45°, which indicated a strong P-limitation was present in soil microorganisms after vegetation restoration (**Figure 2B**). Vector length and vector angle exhibited a significant negative correlation, and microbial P-limitation was switched to microbial N-limitation with C limitation increasing (**Figure 2C**). As shown by enzyme stoichiometry, soil microorganisms were all C-limited. C and N co-limitation on AC was shifted to C and P co-limitation through vegetation restoration (**Figure 2D**).

For soil stoichiometric homeostasis, the linear fit between Ln(C:N) and Ln(C:N_mic_) was significant with *1/H* of 1.18 (**Figure 2E**), indicating that soil C:N_mic_ was sensitive to changes in soil C:N. By comparison, the linear fit between Ln(C:P) and Ln(C:P_mic_) was not significant (**Figure 2F**), indicating that soil microbial C:P_mic_ was strongly stable during vegetation restoration.

### 3.4 Soil ecosystem multifunctionality

**Figure 3:**
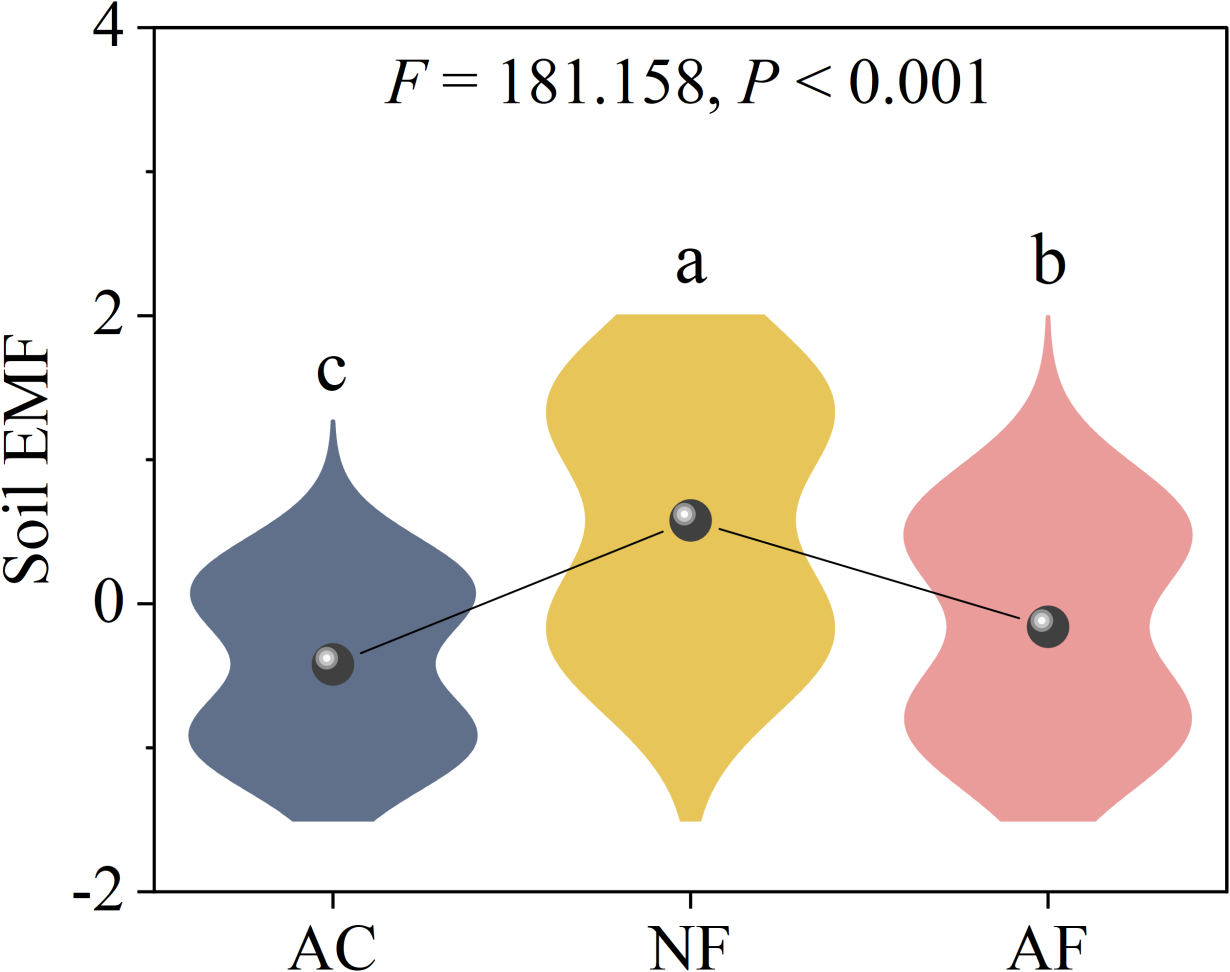
Effect of vegetation restoration on soil ecosystem multifunctionality in abandoned cropland. AC, abandoned cropland; NF, naturally restored secondary forest; AF, artificial planted forest. Different lowercase letters indicated significant differences between vegetation restoration types (LSD, *P***<** 0.05).

**Figure 4:**
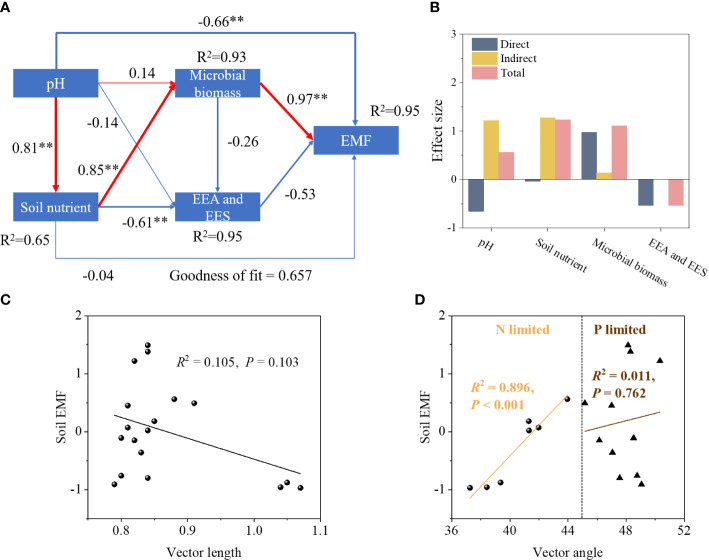
The correlation between soil factors, vector characteristics and soil EMF. **(A)** PLS-PM model; **(B)** effect size; **(C)** vector length; **(D)** vector angle. EMF, ecosystem multifunctionality. The red and blue lines indicate positive and negative effects, respectively. **P **<** 0.01.

Vegetation restoration significantly increased soil EMF, with NF soils exhibiting the highest EMF compared to AC and AF soils (**Figure 3**). PLS-PM revealed pH and microbial biomass had significant effect on soil EMF (**Figure 4A**). The variation in soil EMF was directly driven by pH, soil nutrient, microbial biomass and EEA with path coefficient of -0.66, -0.04, 0.97, and -0.53, respectively (**Figure 4B**). Moreover, soil nutrient and microbial biomass had higher effects on soil EMF with 1.23 and 1.10 of total standardized effect. EMF was not significantly correlated with vector length, indicating that EMF was not affected by soil C limitation (*P =* 0.103; **Figure 4C**). EMF had a significant linear correlation with vector angle under N limitation (*P<* 0.001) but did not under P limitation (*P =* 0.762; **Figure 4D**), indicating that soil N limitation primarily influenced EMF.

### 3.5 Relationship between soil properties, microbial biomass, and EES

**Figure 5:**
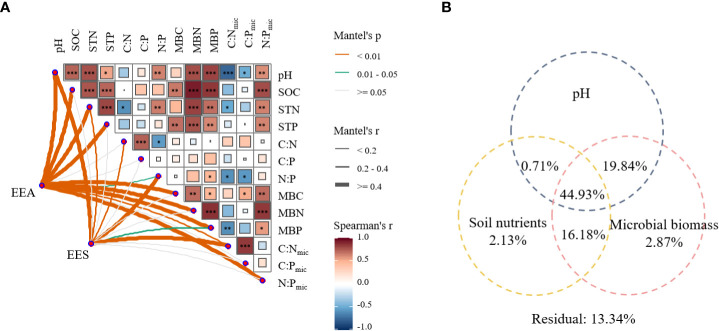
Relationship between pH, soil nutrient, microbial biomass, and soil enzyme characteristics **(A)**, and variation partitioning analysis (VPA) of pH, soil nutrients, and microbial biomass for EEA and EES **(B)**. SOC, soil organic carbon; STN, soil total nitrogen; STP, soil total phosphorus; C:N, ratio of SOC to STN; C:P, ratio of SOC to STP; N:P, ratio of STN to STP; MBC, microbial carbon biomass; MBN, microbial biomass nitrogen; MBP, microbial phosphorus biomass; C:N_mic_, ratio of MBC to MBN; C:P_mic_, ratio of MBC to MBP; N:P_mic_, ratio of MBN to MBP. C_EEA_, the sum of BG, CBH, and BX; N_EEA_, the sum of NAG and LAP; P_EEA_, AP activity. Explanation rates**<** 0 is not shown. *P **<** 0.05; **P **<** 0.01; ***P **<** 0.001.

The Mantel test showed that EEA and EES significantly correlated with most soil nutrients and microbial biomass (**Figure 5A**). EEA was positively correlated with pH, total CNP contents, microbial biomass CNP and their stoichiometries, but negatively correlated with N:P. EES was positively correlated with pH, STN, C:N, N:P, MBC, MBN, and C:N_mic_, but significantly and negatively correlated with MBP.

The VPA revealed that pH, soil nutrients, and microbial biomass jointly accounted for 86.66% of the variation of EEA and EES (**Figure 5B**). In comparison, soil nutrients and microbial biomass accounted for 2.13% and 2.87% of the total variation, respectively. Additionally, the changes in EEA and EES were explained (44.93%) greatly by their interaction.

## 4 Discussion

### 4.1 Effect of vegetation restoration on soil EEA and EES

Vegetation restoration has commonly been shown to alter vegetation, soil properties and soil enzyme activities (Deng et al., 2019; Yang et al., 2020). During the process of vegetation restoration, vegetation and soil components are slightly linked, coevolved vegetation-soil interactions can significantly influence ecological processes (Xiao et al., 2021). In this study, we found vegetation restoration did not alter or significantly reduce soil C- and N- related enzyme activities due to the presence of large amounts of vegetation litters in NF and AF soils, whose decomposition resulted in the return of large amounts of C and N nutrients to the soil. This indicated that the vegetation restoration allowed to meet the N-uptake needs of microorganisms by inputting sufficient C and N in the form of litter. Contrary to C- and N- related enzyme, higher P-related enzyme activity was detected in soils of vegetation restoration compared with AC. The bioavailable P for plant and microbial uptake is mainly derived from phosphate decomposition. As vegetation restoration proceeds, the organic acids secreted by plant roots and extracellular enzymes secreted by microorganisms promote soil P content and availability (Xu et al., 2017; Zhu et al., 2021). However, a large amount of soil P is absorbed by the plant, weakening microbial uptake of soil P. Soil P content is not sufficient to simultaneously meet the P uptake requirements of plants and microorganisms, leading to competition between plants and microorganisms for P uptake. Thus, microorganisms use more energy to increase relative input to produce P- related enzymes. The differences in C/N and P- related enzyme changes after vegetation restoration, indicating microorganisms can adapt their physiological and biochemical characteristics to environmental changes by altering the EEA (Sinsabaugh et al., 2008; Deng et al., 2019).

### 4.2 Effect of vegetation restoration on soil microbial metabolic limitation

Vegetation restoration increases vegetation diversity and soil nutrient uptake by plant communities, which may result in a deficit in microbially available nutrients (Deng et al., 2019; Guo et al., 2022). An excellent approach to gain deep insight into microbial metabolic limitation is to quantify the relative investment in C, N, and P acquisition, which can be calculated using the vector characteristics of C-, N-, and P-related enzymes (Moorhead et al., 2016; Gai et al., 2021). Our study showed vegetation restoration caused a slight decrease in vector length but a significant increase in vector angle (> 45°, **Figure 2B**). This indicated that the mineralised decomposition of litter during vegetation restoration alleviated the energy requirement of microbial metabolic activities to some extent but increased the P requirement. Although vegetation restoration increased soil P content, plants were usually more efficient at nutrient uptake than microorganisms (Lemanski et al., 2019), and microorganisms were more likely to be P-limitation. The scatter plot of N_EEA_ : P_EEA_ vs. C_EEA_ : N_EEA_ (**Figure 2D**) also indicated that microorganisms in this region are chronically C-limited and shifted from N- to P- limitation after vegetation restoration. The increase in plant biomass intensifies plant competition with microorganisms for P uptake. Accordingly, microorganisms need to consume more energy to synthesize P-related enzymes, resulting in C and P co-limitation. Naturally restored secondary forests lead to the most P- limitation of microorganisms owing to high plant abundance and diversity (Cardinale et al., 2012).

Analysis revealed that soil nutrients (i.e. SOC, STN, and STP) had a significant correlation with microbial biomass (i.e. MBC, MBN, and MBP) (**Figure 5A**), suggesting a strong coupling between soil nutrients and microbial biomass during vegetation restoration. Soil microorganisms could adapt to changes in soil nutrients by regulating nutrient use strategies through homeostasis (Gai et al., 2021). Homeostasis theory argues that living organisms can maintain the elemental composition within a narrow range rather than undergoing drastic changes in response to changes in the external environment (Sardans et al., 2012). Therefore, the degree of adaptation of microorganisms in response to environmental changes can be expressed by the strength of homeostasis (Yu et al., 2010). During vegetation restoration, vegetation coverage may affect the regulation of homeostasis (Cleveland and Liptzin, 2007; Zhou et al., 2018a). Our research revealed C:P_mic_ was strongly homeostatic, indicating microorganisms can regulate their metabolic activity in response to vegetation restoration, and the series of physiological adjustments can be reflected in the secretion of C- and P- related enzymes. According to the resource allocation model, prompted microorganisms to use more energy for P-acquiring enzyme synthesis (Soares and Rousk, 2019). This result led to a strong positive relationship between C and P uptake. Microorganisms can stabilise internal C and P by regulating their nutrient balance. C:N_mic_ was also homeostatic but not as strongly as C:P_mic_, because microorganisms do not strictly regulate their internal N content, and thus microbial N is more susceptible to changes in soil environment, suggesting that strengthening soil N management in the soil degradation area of China Danxia may be more beneficial to ecosystem restoration.

### 4.3 Effect of vegetation restoration on soil ecosystem multifunctionality

The improvement of soil EMF was aided by vegetation regeneration (**Figure 3**). Indicating that higher soil nutrient content and availability encouraged soil microbial proliferation, accelerated the geochemical cycling of soil nutrients, and ultimately improved soil ecological functions, soil EMF was significantly associated with soil nutrients, microbial biomass, and EEA (**Figure 4A**, **Figure S1**) (Fry et al., 2018; Liu et al., 2021; Ma et al., 2022). Soil nutrients greatly influenced how soil enzyme response to vegetation restoration (**Figure 4A**; Guo et al., 2022). Inversely, vegetation restoration can also improve soil nutrient use efficiency by increasing EEA. In addition, vegetation restoration increases plant population diversity (in terms of abundance and functional group richness), indirectly affecting soil EMF (Segura et al., 2021). Soil EMF was not significantly correlated with C- limitation (**Figure 4C**), indicating that C- limitation was not a major influencing factor of soil EMF in the study region despite microbial C- limitation. Soil EMF was significantly correlated with N- limitation, but not with P- limitation (**Figure 4D**). These findings indicate that although soil EMF was strongly correlated with soil nutrients, soil N instead of soil C and P was the key influencing factor of regional soil EMF (**Table S2**). Ma et al. (2022) also observed that soil EMF was mainly subject to soil N content. Compared to AF, NF significantly increased soil carbon input resulting in improvement of N benefits, which stimulated soil microbial activity (**Table 1**; Peng et al., 2021), further improving soil geochemical cycling and EMF. In addition, higher species diversity in natural ecological succession benefits soil ecosystem stability (Cardinale et al., 2012).

### 4.4 Drivers of soil EEA and EES

VPA showed that pH (25.26%), soil nutrients (23.42%), and microbial biomass (32.99%) accounted for most of the variation of EES (**Figure 5B**). Microbial biomass accounted for a larger proportion than pH and soil nutrients, likely because of the direct involvement of soil microorganisms in decomposing organic matter and stimulating EEA (Shi et al., 2018; Moore et al., 2020). Furthermore, vegetation restoration resulted in changing environmental conditions, such as increased soil nutrient input and microbial biomass, which greatly enhanced microbial metabolic activities (**Figure 5**) and ultimately promoted extracellular enzyme synthesis. The results of this study fit into the theoretical framework of soil EES, and microbial nutrient limitation addressed by Yang et al. (2020). Vegetation restoration of abandoned cropland caused an increase in plant root biomass and soil EMF with an enhancement of degradative enzyme activity (Xu et al., 2017; Moore et al., 2020), leading to C and N mineralization to mitigate N limitation in the early stage of restoration. The study region is in a subtropical monsoon climate zone with strong soil leaching and weathering. Soil P is at risk of being lost and soils tend to exhibit P deficiency (Vitousek et al., 2010). As vegetation restoration proceeds, the N limitation is gradually lifted due to the increasing abundance of N materials. However, because soil P is derived from a limited number of types of sources, there is competition between microorganisms and plants for P nutrients. This is especially obvious for NF soils, where the higher vegetation abundance and diversity leads to more severe microbial P limitation.

pH also accounted for a high percentage of the variation of soil EES, suggesting that pH has an important effect on enzyme activity (Kivlin and Treseder, 2013). As the vegetation restoration proceeded, soil pH gradually shifted from strongly acidic to weakly acidic pH, which increased soil microbial abundance and activity and facilitated the geochemical cycling of soil N (Kemmitt et al., 2006), leading to a significant increase in soil N content. Zhou et al. (2018b) showed that soil pH is a key factor affecting P availability in red soils and that a pH of at least 5.0 should be targeted to increase P availability. Therefore, microorganisms in NF (pH 6.15–6.28) and AF (pH 5.91–6.01) soils need to synthesize more P-acquiring enzymes to meet microbial P requirement. The abandoned cropland in the soil degradation area of the China Danxia in this study was N-limited but shifted from microbial N- to P- limitation as vegetation restoration proceeded. More importantly, while both microbial biomass and pH accounted for the variation in EES well, the interaction of pH, soil nutrients, and microbial biomass accounted for the largest variation. Therefore, biotic and abiotic factors should be considered when investigating microbial nutrient limitations during ecological restoration. The findings in this study may help to develop a strategy for nutrient management and soil ecosystem in the ecological degradation area of the China Danxia and provide a basis for understanding microbial nutrient limitation in other ecologically degraded areas during their ecological restoration.

## 5 Conclusions

Our research explored microbial nutrient limitation and soil ecosystem multifunctionality with vegetation restoration from the perspective of microbial metabolisms in the China Danxia. Soil EEA and associated stoichiometries were significantly affected by vegetation restoration. Vector analysis showed microbial metabolisms from N-limitation to P-limitation with enzyme N:P less than 1 after vegetation restoration, especially in soil of naturally restored secondary forests. Moreover, soils of vegetation restoration could require less C-resources for soil microorganisms with lower vector length. Vegetation restoration relieved the soil N-limitation and improved soil N nutrient, and thus promoted soil ecosystem multifunctionality. Furthermore, the key factor affecting soil microbial nutrient limitation was the interaction of abiotic (pH and nutrients) and biotic factors, and soil ecosystem multifunctionality was affected as well. Our findings can provide insight into understanding of microbial metabolisms of nutrient cycling, which is benefit for developing ecological restoration on nutrient management strategies and soil ecosystem.

## Data availability statement

The original contributions presented in the study are included in the article/**Supplementary Material**. Further inquiries can be directed to the corresponding author.

## Author contributions

CW: Methodology, Validation, Formal analysis, Visualization, Writing-original draft; QY: Conceptualization; CZ: Investigation, Writing-review & editing, Funding acquisition; XZ: Methodology, Formal analysis; JC: Methodology, Formal analysis; KL: Conceptualization, Writing-review & editing, Funding acquisition. All authors contributed to the article and approved the submitted version.
