# Supplementary material for: Vegetation restoration of abandoned cropland improves soil ecosystem multifunctionality through alleviating nitrogen-limitation in the China Danxia
Source: Front Plant Sci. 2023 Feb 28;14:1116179. doi: 10.3389/fpls.2023.1116179 (PMC10011436; doi:10.3389/fpls.2023.1116179)
Supplement: Supplementary file 2 [file DataSheet_2.docx]

Supplementary Material

Vegetation restoration of abandoned cropland improves soil ecosystem multifunctionality through alleviating nitrogen-limitation in the China Danxia

Chao Wang, Qiannan Yang, Chi Zhang, Xiaolong Zhang, Jing Chen, Kexue Liu

*** Correspondence:** Kexue Liu 28257448@qq.com

# Supplementary Figures and Tables

## Supplementary Tables

### Table S1 Commission number, abbreviation, and corresponding substrate of soil extracellular enzymes

| **Soil extracellular enzyme** | **Abbreviation** | **International classification number** | **Corresponding substrate** |
| --- | --- | --- | --- |
| β-1,4-glucosidase | BG | EC 3.2.1.21 | 4-MUB-β-D-glucoside |
| β-Dcellobiohydrolase | CBH | EC 3.2.1.91 | 4-MUB-β-D-cellobioside |
| Xylosidase | BX | EC 3.2.1.37 | 4-MUB-β-D-xyloside |
| β-N-acetylglucosaminidase | NAG | EC 3.1.6.1 | 4-MUB-N-acetyl-β-D-glucosaminide |
| Leucine aminopeptidase | LAP | EC 3.4.11.1 | L-leucine-7-amido-4-methylcoumarin hydrochloride |
| Alkaline phosphatase | AP | EC 3.1.3.1 | 4-MUB-phosphate |

### Table S2 The relationship between soil abiotic-biotic factors and EMF, and their deviation explained to EMF

| **Response variable** | **Parameter** | **Regression equation** | **P-value** | **Deviation explained** |
| --- | --- | --- | --- | --- |
| Regression |  | *y* = -2.34 + 0.*05x*_MNB_ + 0.*01x*_NEEA_ |  |  |
| EMF | MBN and N_EEA_ |  | < 0.001 | 96.16 (*R*^2^) |
| Explained rate |  |  |  |  |
| EMF | MBN |  | 0.82 | 37.49 |
|  | N_EEA_ |  | 0.04 | 17.21 |
|  | Interactions |  | 0.31 | 41.49 |

MBN, microbial biomass nitrogen; EMF, soil ecosystem multifunctionality. N_EEA_, the sum of NAG and LAP.

## Supplementary Figures

**Supplementary Figure 1.** The relationship between soil nutrients, microbial biomass, extracellular enzyme activities and soil EMF. SOC, soil organic carbon; STN, soil total nitrogen; STP, soil total phosphorus; MBC, microbial biomass carbon; MBN, microbial biomass nitrogen; MBP, microbial biomass phosphorus; MBP, microbial biomass phosphorus; C_EEA_, the sum of BG, CBH, and BX; N_EEA_, the sum of NAG and LAP; P_EEA_, AP activity. *, P < 0.05; **, P < 0.01.
