# Supplementary figures and images for: Vegetation restoration of abandoned cropland improves soil ecosystem multifunctionality through alleviating nitrogen-limitation in the China Danxia
Source: Front Plant Sci. 2023 Feb 28;14:1116179. doi: 10.3389/fpls.2023.1116179 (PMC10011436; doi:10.3389/fpls.2023.1116179)

## Microbial nutrient limitation and EMF

+ Increased  
- Decreased

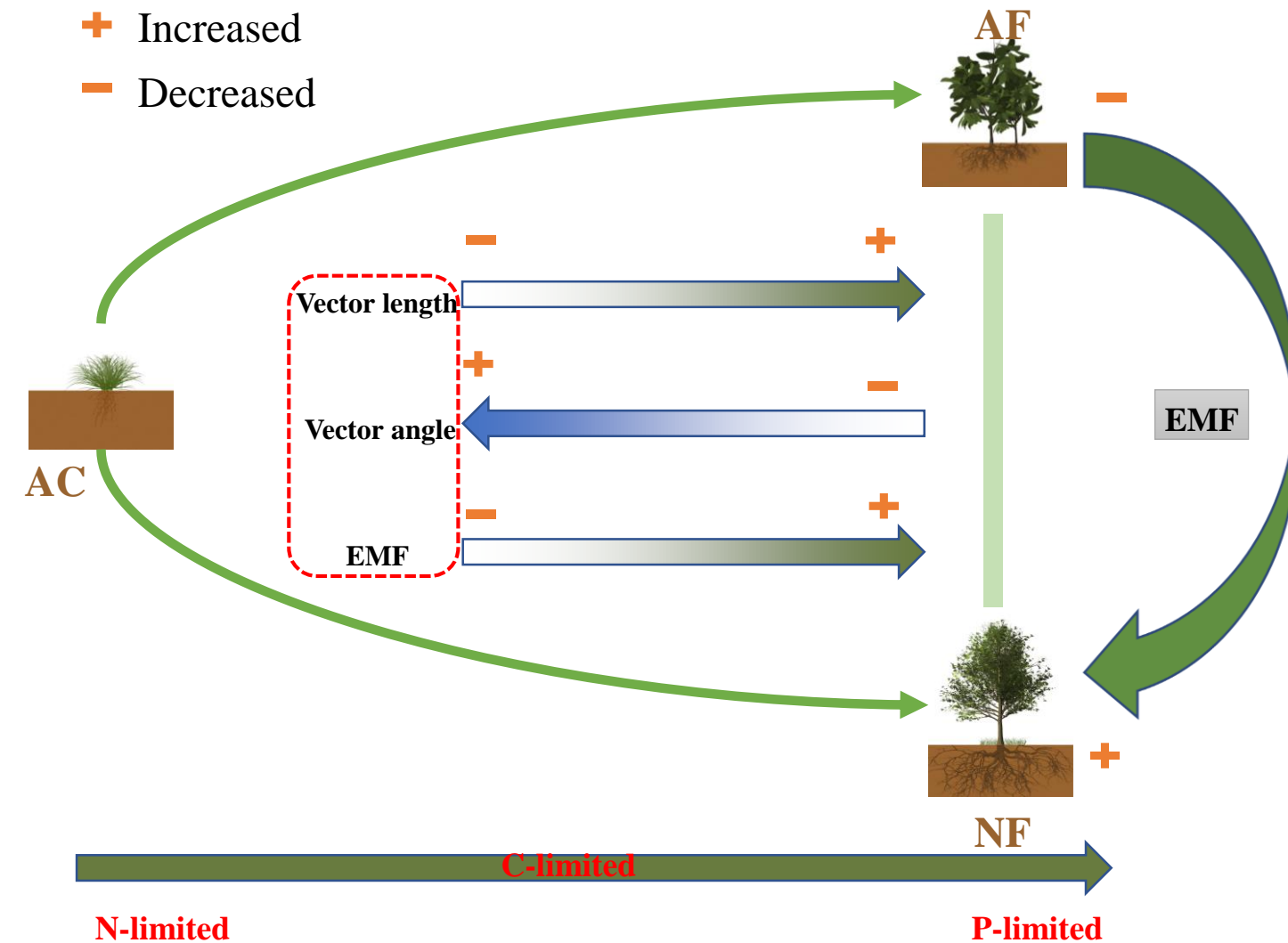

## Influence factors

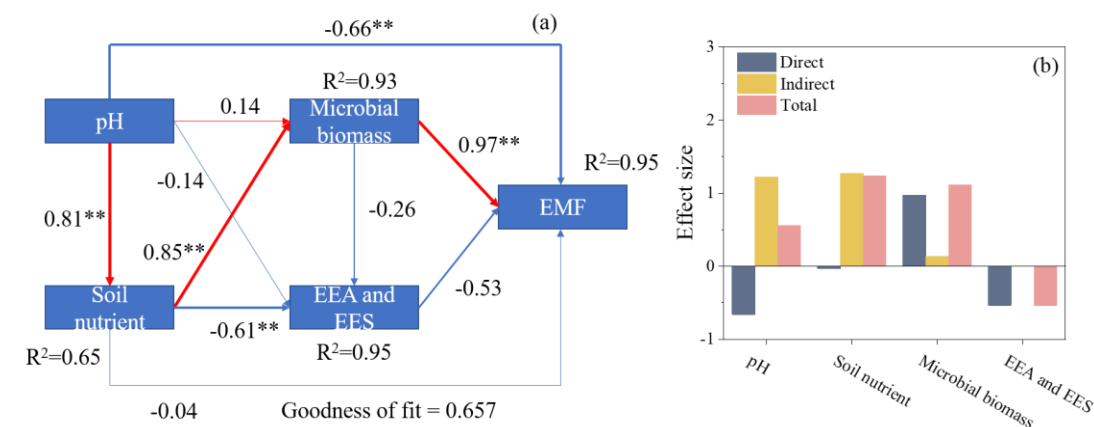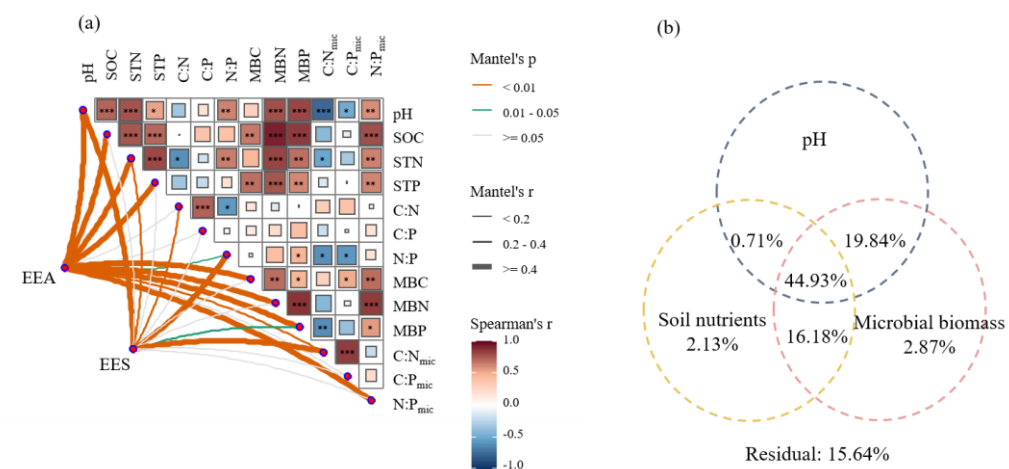

Supplement: Supplementary file 3 [file Image_1.pdf]
